# The Book World of Medicine and Science

**Published:** 1896-06-06

**Authors:** 


### The Book World of Medicine and Science.

Some Prolegomena to a Philosophy of Medicine. By Giles F. Goldsbrough, M.D. (London: John Bale and Son.)

One of the uses of error is the clarification of truth. When a pronounced homœopathist publishes the data out of which a philosophy of medicine may be constructed, we may take it for granted that he does his best to give his readers the very soul and essence and inner reality of the principle or principles which are to constitute the life-blood of the intellectual organism he designs to create. In other words, we shall do homœopathy and homœopathists no wrong if we take Dr. Goldsbrough's book and try as fairly as may be possible to set forth its why, its wherefore, and its whither.

"A philosophy of medicine"! The phrase reads well. But when one comes to ask oneself the question, what precisely may it mean? the answer does not spring all at once, like Minerva, full-armed from the head of Jove. Now, in medicine, if anywhere, it is necessary to be absolutely precise. Not only must one have a meaning, but we must know exactly what that meaning is. Take a parallelism. In matters financial precision is of the very essence of business. The financier, the accountant, are exact to the minutest fraction. Only on this condition is business possible. But if that be so in the matter of finance, which after all, is only one of the adjuncts of life, how much more must exactitude be imperatively necessary in matters which directly concern life itself? What one feels is that philosophising about medicine, if the philosophising be not kept sternly in hand, is apt to degenerate into solemn trifling.

Is a philosophy of medicine possible? No doubt it is! But what must it include? It must surely include facts; a reasoned exposition of facts; and a method or methods. What does Dr. Goldsbrough, a typical representative of homœopathy, give us instead of these? Facts he certainly gives; though they are facts mostly without special relevance. As for a reasoned exposition, it is nowhere attempted; whilst of practical methods hardly as much as a single mention is made. The title of Dr. Goldsbrough's book, "A Philosophy of Medicine," seems, indeed, to have been selected entirely on the *lucus a non lucendo* principle, for of medicine it contains little, and of philosophy none at all.

There are sixty-six closely-printed octavo pages in the book, and in that amount of space it should have been possible at least to indicate the outlines of a general scheme of medicine according to the principles of homœopathy. But the net result of reading those pages with the utmost possible care is a condition of absolute mental bewilderment. Nowhere can anything precise be so much as approached. There is, indeed, some affirmation of what is called a "law of similars," but if we had not had some previous knowledge of what homœopaths mean by a "law of similars," it would have been quite impossible to extract any definite meaning from Dr. Goldsbrough's pages. So far as can be made out, Dr. Goldsbrough means by a "law of similars" this—that those physiological and pathological conditions which in health may be induced by certain substances, in disease may be cured by the same or similar substances. Now, everybody will admit that, to say the least, there is a tremendous amount of *a priori* improbability about such a "law" as this; and if Dr. Goldsbrough had wished to base a "philosophy" upon it, the least he could have done would have been to expound its significance clearly, and to offer some sort of demonstration, in fact and in theory, of its certainty, or at any rate, of its high probability. But, as we have said, of this one and only principle which the book contains, there is no exposition at all, whilst of supporting facts and reasoned demonstration there is not so much as even a shadow.

Readers may, not unnaturally, ask us for proofs of these assertions. The proof will be found in Dr. Goldsbrough's book itself. But by way of showing the curious what they have to expect, the following passages, taken almost at random from scores of similar ones, are transcribed. Says Dr. Goldsbrough, p. 4, "As far as average science is concerned, homœopathy is therefore ultra-rational—it is beyond science." Now what does this mean? Does it mean anything at all? And if it means anything at all, does it not mean, at any rate to the practical professor of the art and industry of healing, something entirely preposterous? Here is another specimen of Dr. Goldsbrough's science and philosophy. He says, p. 13: "In a series of prolegomena to a philosophy of medicine it must first be noticed what are the special features in medical knowledge which distinguish it as knowledge from the everyday knowledge of the people at large? Broadly stated, this distinction consists in the difference carried as far as possible between positive science and common sense." Well, that may be the case with homœopathy; it may be, and probably is the fact, that homœopathy and common sense are entirely antipodal to each other; but we altogether decline to admit that "broadly speaking, the distinction between medical knowledge and common knowledge is the same as the distinction between positive science and common sense"; and for this reason, we decline to believe that there is any true distinction at all between positive science and common sense. Positive science is common sense, the very essence of common sense; and whoever tries to make a distinction between these two lays himself open to the shrewd suspicion that he has very little kinship with either the one or the other. It may be retorted that Dr. Goldsbrough has not made his meaning quite clear; that, in short, he means something different from what he says. But what has a man to do with "philosophising" if he has not attained the elementary art of setting forth his meaning with clearness and precision?

What position, then, does homœopathy, that is, honest homœopathy, occupy in relation to the general progress of medicine? It occupies the position which medicine generally occupied before it was brought within the operation of the methods of inductive science. Homœopathy is a crude survival in Hahnemann and his followers of the methods of the pre-Baconian period. So far as it is concerned, Bacon lived and wrote in vain. The reproach which Bacon uttered against the medicine of his time was that it sought in speculation and abstract theorising the knowledge which could only be gained in the physiological laboratory and at the bedside. That reproach scientific medicine has long since wiped away. That reproach is still the opprobrium of homœopathy. As Dr. Goldsbrough himself admits, "Homœopathy is ultra-rational; it is beyond science." In a word, in the general progress of medicine homœopathy is to scientific medicine what the crude gooseberry is to the ripe fruit; or what the ignorant and elementary preacher of the Salvation Army is to the learned expositor who brings all the best methods of literary and scientific culture to bear upon the infinitely difficult problems he is called upon to elucidate and to commend to the sincere acceptance of his more or less critical fellow-men. *Ex nihilo nihil fit.* Homœopathy begins in the abstract and ends in the inane.

